# Olfactory Receptors in Non-Chemosensory Organs: The Nervous System in Health and Disease

**DOI:** 10.3389/fnagi.2016.00163

**Published:** 2016-07-05

**Authors:** Isidro Ferrer, Paula Garcia-Esparcia, Margarita Carmona, Eva Carro, Eleonora Aronica, Gabor G. Kovacs, Alice Grison, Stefano Gustincich

**Affiliations:** ^1^Institute of Neuropathology, Bellvitge University Hospital, Hospitalet de Llobregat, University of BarcelonaBarcelona, Spain; ^2^Center for Biomedical Research in Neurodegenerative Diseases (CIBERNED)Madrid, Spain; ^3^Bellvitge Biomedical Research Institute (IDIBELL), Hospitalet de LlobregatBarcelona, Spain; ^4^Neuroscience Group, Research Institute HospitalMadrid, Spain; ^5^Department of Neuropathology, Academic Medical Center, University of AmsterdamAmsterdam, Netherlands; ^6^Institute of Neurology, Medical University of ViennaVienna, Austria; ^7^Scuola Internazionale Superiore di Studi Avanzati (SISSA), Area of NeuroscienceTrieste, Italy

**Keywords:** olfactory receptors, taste receptors, brain, Alzheimer, Parkinson, progressive supranuclear palsy, Creutzfeldt-Jakob disease, schizophrenia

## Abstract

Olfactory receptors (ORs) and down-stream functional signaling molecules adenylyl cyclase 3 (AC3), olfactory G protein α subunit (Gαolf), OR transporters receptor transporter proteins 1 and 2 (RTP1 and RTP2), receptor expression enhancing protein 1 (REEP1), and UDP-glucuronosyltransferases (UGTs) are expressed in neurons of the human and murine central nervous system (CNS). *In vitro* studies have shown that these receptors react to external stimuli and therefore are equipped to be functional. However, ORs are not directly related to the detection of odors. Several molecules delivered from the blood, cerebrospinal fluid, neighboring local neurons and glial cells, distant cells through the extracellular space, and the cells’ own self-regulating internal homeostasis can be postulated as possible ligands. Moreover, a single neuron outside the olfactory epithelium expresses more than one receptor, and the mechanism of transcriptional regulation may be different in olfactory epithelia and brain neurons. *OR* gene expression is altered in several neurodegenerative diseases including Parkinson’s disease (PD), Alzheimer’s disease (AD), progressive supranuclear palsy (PSP) and sporadic Creutzfeldt-Jakob disease (sCJD) subtypes MM1 and VV2 with disease-, region- and subtype-specific patterns. Altered gene expression is also observed in the prefrontal cortex in schizophrenia with a major but not total influence of chlorpromazine treatment. Preliminary parallel observations have also shown the presence of taste receptors (TASRs), mainly of the bitter taste family, in the mammalian brain, whose function is not related to taste. TASRs in brain are also abnormally regulated in neurodegenerative diseases. These seminal observations point to the need for further studies on ORs and TASRs chemoreceptors in the mammalian brain.

## Introduction

Chemical signals acting on appropriate receptors are the most common and precise form of communication. This applies both unicellular organisms with their milieu and to complex individuals in distinct cell types and systems within the same individual. Odorant and pheromone receptors are specialized chemical receptors designed to recognize and respond to volatile molecules by activating down-stream pathways to modulate specific behaviors. This type of receptor includes most of genome in the insects and mammals, and the number of putative ligands is not known but is estimated to be in the thousands. Odorant and pheromone signaling mediates chemical communication among species and among individuals in the same species. This information is crucial in natural settings, enabling individuals to obtain appropriate data about accessibility of food, and to reject toxic and damaging substances; in determining the sexual status of females, permitting mating and reproduction, breeding and nursing; in imprinting; in delimitation of territories and homing; and in attraction, repulsion, perception of danger and navigation. (Hanson, 1999; Ryan, 2002; Blomquist and Vogt, 2003; Doty, 2003; Wyatt, 2003; Brennan and Zufall, 2006; Brewer et al., 2006; Hallem et al., 2006; Wilson and Stevenson, 2006; Menini, 2009; Ihara et al., 2013; Liberles, 2014; Barish and Volkan, 2015; Li and Liberles, 2015; Wicher, 2015).

Odorant or olfactory receptors (ORs) are localized in sensory organs such as the olfactory epithelium in the nasal cavity in mammals, but overwhelming evidence in recent years has shown that the same type of receptors are distributed in many different organs and systems in mammals. These have been called “ectopic olfactory receptors” with distinct but organ- and tissue-specific characteristics. ORs and down-stream and related signaling molecules are also present in non-olfactory regions of the nervous system. Moreover, they are vulnerable to several neurodegenerative and mental diseases, and to drugs.

## Olfactory Receptors (ORs), Down-Stream Effectors, Transporters and Related Olfactory-Signalling Helpers in Chemosensory Organs

Olfactory or odorant receptors (ORs) have been identified as members of the G-protein-coupled receptors (GPCRs) family which share characteristic conserved trans-membrane motifs (Buck and Axel, 1991; Kato and Touhara, 2009; Malnic et al., 2010). *OR* genes constitute the largest family of GPCRs with about 1000 genes in the mouse and about 370 in humans (Buck and Axel, 1991; Glusman et al., 2000a,b; Zozulya et al., 2001; Young et al., 2002; Zhang and Firestein, 2002; Zhang et al., 2004, 2007; Niimura and Nei, 2003, 2005; Malnic et al., 2004; Olender et al., 2004; Kanageswaran et al., 2015). Amphioxi express ORs similar to those found in advanced vertebrates (Niimura, 2012). In later natural history, ORs diversified and two main classes of ORs appeared during the evolution of Chordata: teleost fish have class I which permits the detection of water-soluble odorants, whereas amphibians, reptiles, birds and mammals developed class II specialized in the perception of aerial odorants (Niimura, 2012; Hoover, 2013). Coelacanth, which is in transition between fish and tetrapods has class I ORs but also seven class II *OR* genes (Picone et al., 2014). The majority of ORs in mammals are classified as class II but between 10% and 20% are class I (Glusman et al., 2001; Zhang and Firestein, 2002; Niimura and Nei, 2003, 2005).

The repertoire of OR expression shows differences among species, not only between clearly separated species such as mice and humans but also among primates including *Homo sapiens* (Rouquier et al., 1998, 2000; Gimelbrant et al., 2001, 2004; Glusman et al., 2001; Godfrey et al., 2004; Gilad et al., 2005; Quignon et al., 2005; Go and Niimura, 2008). African elephants express the largest number of ORs, approximately 2000 *OR* genes and 2000 *OR* pseudogenes, about double the numbers for cows and horses, and a large number when compared with 1000–1200 *OR* genes in common rodents and around 400 *OR* genes in most primates including humans (Niimura et al., 2014). Although no similar data are available for Asian elephants, these animals have an extraordinary capacity to perceive odor differences (Rizvanovic et al., 2013). The reasons for OR expression changes during mammalian evolution are not known but they are most probably related to environmental and practical factors. For example, dolphins have reduced numbers of ORs but frugivorous bats among Chiropterans have developed particular OR profiles adapted to their diet (Hayden et al., 2014). Among primates, not only ORs but also the expression of V1Rs has been modified during evolution when compared with other mammals (Yoder and Larsen, 2014). The study of the cranium in *Neanderthals* has led to the suggestion that those hominids had better olfactory capacity than present humans (Hughes et al., 2014). Finally, to add more complexity, the expression of some genes is subjected to individual variations in a given species (Young et al., 2003).

Activation of ORs leads to the dissociation of the Olfactory G-protein Golf into three subunits: α, β and γ (Li et al., 2013a). olfactory G protein α subunit (Gαolf), Gβ1 and Gγ13 form the functional heterotrimeric G protein in olfactory sensory neurons (Li et al., 2013a). Ric-8B co-localizes and interacts with Gαolf and γ13 in the cilia of olfactory sensory neurons and mediates signaling of ORs (Von Dannecker et al., 2005, 2006; Kerr et al., 2008).

Activation of Gαolf and adenylyl cyclase 3 (AC3) induces an increase in the intracellular concentration of cyclic adenosine monophosphate (cAMP), activation of cAMP-gated channel, external Ca^++^ influx, and membrane depolarization (Pace et al., 1985; Sklar et al., 1986; Nakamura and Gold, 1987; Jones and Reed, 1989; Lowe et al., 1989; Bakalyar and Reed, 1990; Dhallan et al., 1990; Firestein et al., 1991; Liman and Buck, 1994; Bönigk et al., 1999; Mombaerts, 1999, 2004; Firestein, 2001; Stephan et al., 2009; Billig et al., 2011). General anosmia is produced by a targeted disruption of AC3 in mice (Brunet et al., 1996; Wong et al., 2000). Mice null for the expression of Gαolf do not perceive odors (Belluscio et al., 1998; Luo et al., 2002); conditional Gγ13 knockout mice exhibit altered recognition of several odorants (Li et al., 2013b). More subtle regulation occurs following modulation of βγ subunits of Golf complex which suppress the expression of several odor-related genes, probably through the methylation of specific histones (Ferreira et al., 2014).

Repolarization occurs by extruding intracellular Ca^++^ through the plasma membrane with the coordinated action of a non-potassium-dependent sodium/calcium exchanger which is modulated by the olfactory marker protein (OMP), a potassium-dependent sodium/calcium exchanger, and a plasma membrane Ca^++^-ATPase (Pyrski et al., 2007; Kwon et al., 2009; Saidu et al., 2009; Stephan et al., 2011).

ORs and other chemoreceptors require specific cofactors to improve the membrane localization of the receptors (Jungnickel et al., 2001; Saito et al., 2004; Behrens et al., 2006). Co-factors of GPCRs upon binding to specific domains of receptors, facilitate or inhibit cell surface expression (Duvernay et al., 2004, 2009; Hague et al., 2004; Syme et al., 2006). Receptor transporter proteins 1 and 2 (RTP1 and RTP2) and receptor expression enhancing protein 1 (REEP1) enhance OR function by the recruitment of ORs to lipid rafts (Saito et al., 2004). Recently a complex interaction between transient receptor potential 2 channel (TRPC2), which establishes the first electrical signal in the pheromone transduction pathway has been discovered in the peripheral olfactory organs of rats (Mast et al., 2010). Homer protein isoforms 1 b/c and 3 bind to proline-rich sequences on proteins associated with calcium signaling (Worley et al., 2007) and RTP1.

UDP-glucuronosyltransferases (UGTs) are localized in the nuclear membrane and endoplasmic reticulum where glucuronidate xenobiotic and endobiotic compounds are present, thus facilitating their excretion and contributing to detoxification of cells (Tukey and Strassburg, 2000; Mackenzie et al., 2005a,b). Various UGTs have been identified in the olfactory epithelium and olfactory bulb (Leclerc et al., 2002; Heydel et al., 2010) where they probably participate in the removal of compounds produced during the process of OR activation and signaling.

OR expression is not fixed; rather, OR presentation is modulated by noradrenergic inputs (Hague et al., 2004; Eckmeier and Shea, 2014) and odorant stimulation (Kim et al., 2015b), among other factors (Malnic et al., 2010). Interestingly, variations of temperature in the olfactory epithelium of Xenopus tadpoles result in variable activation in the olfactory glomeruli (Kludt et al., 2015). Regulation of olfactory organs and receptors has also been observed with high environmental temperatures in *Drosophila* (Martin et al., 2011; Riveron et al., 2013). Similar complementary effects of temperature and odors probably occur in the olfactory system in rodents. Curiously, olfaction thresholds increase in hyperbaric situations (Ay et al., 2014). These examples illustrate the effects of physical environments on smell perception. Finally, behavior and odor memories can modify the expression of chemoreceptors in butterflies and honeybees (Briscoe et al., 2013; Claudianos et al., 2014).

## ORs in Non-Olfactory Tissues and Organs

Growing evidence indicates that the olfactory epithelium is only one of the multiple regions bearing ORs and probably the only organ in which ORs are charged with reacting to diverse small aerial molecules which are processed into odors. Yet the perception of odors (and their recognition in humans) is not the only function of ORs. More readily, activation of certain ORs switches on complex cellular responses mediated by neural networks, neurotransmitters and hormones which in turn determine sophisticated behaviors. ORs located in non-olfactory tissue and organs are chemoreceptors able to trigger complex response receptors but not precisely linked to any sensorial perception.

ORs located in non-chemosensory organs were categorized as “ectopic” ORs because they were expressed in unexpected regions. However, this term is misleading as it implies that “ectopic” ORs might have functions related to olfaction. Yet mounting evidence indicates that the function of “ectopic” ORs has no relation to the capture of odors. Ectopic ORs were first identified in the testes and sperm and their function seems to be related to discriminatory chemotaxis (Parmentier et al., 1992; Walensky et al., 1995, 1998; Asai et al., 1996; Vanderhaeghen et al., 1997; Branscomb et al., 2000; Spehr et al., 2003; Fukuda et al., 2004; Fukuda and Touhara, 2006; Veitinger et al., 2011).

ORs are also expressed in germinal cells (Goto et al., 2001), embryos (Nef and Nef, 1997; Dreyer, 1998), developing heart (Drutel et al., 1995), developing muscle (Griffin et al., 2009) and axons of olfactory neurons. These observations show the participation of ORs in a wide range of processes at several stages of development (Baker et al., 2015) including angiogenesis (Kim et al., 2015a) and direction of nerve fibers in the olfactory bulb (Vassalli et al., 2002; Feinstein and Mombaerts, 2004; Feinstein et al., 2004).

Certain ORs and functional mediators are also expressed in lung (Zhang et al., 2004; Li et al., 2013b; Gu et al., 2014), spleen (Blache et al., 1998), liver (Yuan et al., 2001), gastrointestinal tract (Yuan et al., 2001; Braun et al., 2007), placenta (Itakura et al., 2006), prostate (Xu et al., 2000; Xia et al., 2001; Neuhaus et al., 2009), erythroid cells and peripheral blood cells (Feingold et al., 1999; Zhao et al., 2013) and eye (Pronin et al., 2014), among other tissues and organs (Feldmesser et al., 2006; De la Cruz et al., 2009; Kang and Koo, 2012; Flegel et al., 2013).

ORs and functional signaling molecules are abundant in the kidney (Pluznick et al., 2009; Rajkumar et al., 2014) where they regulate the glomerular filtration rate (Pluznick et al., 2009; Pluznick, 2014).

Recent transcriptome studies using cDNA arrays in the rat have shown expression of ORs and taste receptors (TASRs) in choroid plexus (Quintela et al., 2013). Interestingly, TASR and OR transduction pathways are regulated by hormones, with the expression of transcripts being different in males and females (Quintela et al., 2013). As in other non-sensory regions, ORs in the choroid plexus do not participate in the identification of odors but rather in the detection of soluble molecules in the CSF (Quintela et al., 2013).

Several ORs are expressed in a number of tissues, whereas certain ORs are only expressed in testis (Flegel et al., 2013). OR-specific functions have been identified in a very small number of cases. *Olfr13* (MOR23) participates in the journey of sperm, growth of axonal growth cones and migration of myocytes (Fukuda et al., 2004; Griffin et al., 2009). OR1A and OR2A4 regulate actin cytoskeleton and cytokinesis (Zhang et al., 2012).

ORs responding to short chain fatty acids produced by gut microbiota modulate renin secretion and regulation of blood pressure (Pluznick et al., 2013), while activation of ORs by spicy species in enterochromaffin cells liberates serotonin (Braun et al., 2007; Breer et al., 2012). OR activation in mouse pulmonary macrophages promotes monocyte chemotactic protein-1 production (Li et al., 2013b). ORs in α-cells of pancreatic islets regulate glucagon secretion (Kang et al., 2015). Renal and cardiovascular ORs regulate blood pressure (Pluznick, 2013). Prostate-specific ORs are regulated by a derivative of androstenone (Neuhaus et al., 2009). Complex interactions like those of microbiota-derived products in gut, which activate ORs in gut and kidney, have an effect in the control of blood pressure (Natarajan and Pluznick, 2015). In summary, ORs in non-sensory tissues and organs are not involved in olfaction but rather carry out diverse and unrelated functions. Their effects are local and there is no connection between these receptors and the brain.

## ORs in Non-Sensory Organs of the Nervous System in Mammals

A number of ORs have been found in the cerebral cortex of mice (Otaki et al., 2004). *Olfr110*, *Adcy3* and* Gnal* (*Gαolf*) mRNAs are also expressed in the cerebral cortex at the ages of new born, and at 3, 6 and 12 months. mRNA expression levels do not vary over the first year of life (Ansoleaga et al., 2013).

Gene array studies have revealed expression of ORs in the human brain and mouse dopaminergic neurons of the substantia nigra (Garcia-Esparcia et al., 2013; Grison et al., 2014). This has been further validated by qRT-PCR analysis (Ansoleaga et al., 2013, 2015; Garcia-Esparcia et al., 2013; Grison et al., 2014). Immunohistochemistry in the adult human brain has shown the presence of OR2H2, OR2A4, and OR6K3 in neurons of the neocortex, hippocampus, dentate gyrus, striatum, thalamus, nuclei of the basal forebrain, hypothalamus, nuclei of the brainstem, cerebellar cortex, dentate nucleus and neurons of the spinal cord.

OR immunostaining predominates in the cytoplasm as a granular precipitate and the intensity is to a certain extent dependent on the size of neurons, as the staining of neurons of the motor nuclei of the cranial nerves, Purkinje cells and CA1 neurons is more intense in comparison with that of cerebellar granule cells and neurons of the dentate gyrus. A mainly cytosolic localization of ORs is supported by sub-cellular fractionation analysis (Garcia-Esparcia et al., 2013). In contrast to the olfactory epithelium, several ORs seem to be expressed in a single neuron in the central nervous system (CNS).

Gαolf is expressed in the striatum, where it may couple dopamine D1 receptors and adenosine A2A receptors to the activation of adenylyl cyclase type 5.1 (Corvol et al., 2001; Hervé, 2011). Nevertheless, Gαolf is not restricted to the striatum; rather it is widely distributed, at least, in the human brain, as well as AC3 (Garcia-Esparcia et al., 2013).

RTP1, RTP2, and REEP1 are expressed in the cytoplasm of neurons, as revealed by immunohistochemistry, paralleling the expression of ORs and down-stream signaling molecules in all brain regions (Garcia-Esparcia et al., 2013).

Finally, various UGTs, including UGT1A6, have been localized in the olfactory mucosa and olfactory bulb (Leclerc et al., 2002). Immunohistochemistry reveals UGT1A6 localization in neurons of the human CNS (Garcia-Esparcia et al., 2013).

*Olfr110*, *Adyc3*, and *Gnal* (*Gαolf*) mRNAs are also expressed in the cerebral cortex of mice (Ansoleaga et al., 2013; Grison et al., 2014).

Although co-localization studies are needed to determine whether all the components of the receptor signaling pathway are present in the same cell, *in vitro* studies have identified functional responses of neuronal ORs in the face of external stimuli which cannot be produced in the absence of the signaling pathway. Thus, taking advantage of transgenic labeling, laser capture microdissection coupled to nano cap-analysis of gene expression (nanoCAGE) technology on isolated A9 and A10 cells of the substantia nigra in mice, has revealed that a subset of ORs is expressed in mesencephalic dopaminergic neurons (Grison et al., 2014). OR expression in the mesencephalon has been validated by RT-PCR and *in situ* hybridization.

Heterologous HEK 293 cells expressing *Olfr287* following transfection respond to selected molecules, particularly S- and R-carvones, and decanoic acid. In the same line, cultured isolated dopaminergic neurons are activated by odor-like agonists and carvones as revealed by neuronal Ca^++^ entry upon stimulation. Ca^++^ levels return to normal after washout (Grison et al., 2014).

ORs have also been reported in the autonomic nervous system and murine sensory ganglia (Weber et al., 2002; Manteniotis et al., 2013).

Like ORs in other non-sensory organs and regions, the function of ORs in brain differs from the specific detection of molecules by ORs in the nasal cavity, and it is not related to the perception of odors.

## Olfactory-Binding Proteins and OR Ligands

OBP are small soluble extracellular proteins related to pheromone and odor transduction, mainly but not exclusively found in olfactory organs such as pheromone-binding and odorant-binding proteins. These proteins trap and convey odorants and pheromones to specific receptors (Vogt and Riddiford, 1981; Vogt et al., 1985, 1991; Pevsner et al., 1986, 1988, 1990; Pelosi and Maida, 1990). In mammals, OBPs belong to the super family of carrier protein lipocalins (Flower, 1996) present in the lateral nasal gland and olfactory epithelium (Pevsner et al., 1988; Dal Monte et al., 1991; Paolini et al., 1998). OBPs are also present in several fluids including vaginal discharge, urine, saliva, tears and amniotic fluid (Singer et al., 1986; Garibotti et al., 1995; Redl, 2000; Briand et al., 2004; Guiraudie-Capraz et al., 2005).

Certain lipocalins, such as lipocalin 2, are produced in brain where they have important functions in regulating inflammatory responses, angiogenesis, and astrocyte and microglial activation (Lee et al., 2011; Hamzic et al., 2013; Jin et al., 2014; Nam et al., 2014; Xing et al., 2014; Wu et al., 2015). Apolipoprotein D (APOD), a member of the lipocalin family and transporter of small hydrophobic ligands, is also expressed in brain (Rassart et al., 2000). The close relationship between OBPs and other lipocalins has prompted the hypothesis, that this type of transporter links chemical communication and immunity (Stopková et al., 2014). Therefore, OBPs in brain and other organs do not necessarily participate in olfaction, but rather are used in other metabolic functions which have nothing in common with smell.

Various natural ligands of OBPs have been recognized in mammals (Robertson et al., 1993; Glasgow et al., 1995; Marchese et al., 1998; Briand et al., 2004; Sanz et al., 2005; Le Danvic et al., 2009; Scaloni et al., 2011). These are small molecules of about 200–400 mW related to derived fatty acids, steroids, and terpenoids (Wetzel et al., 1999; Spehr et al., 2003; Mombaerts, 2004; Sanz et al., 2005; Shirokova et al., 2005; Jacquier et al., 2006; Saito et al., 2006; Keller et al., 2007; Khafizov et al., 2007; Malnic, 2007; Schmiedeberg et al., 2007; Hsu et al., 2008; Nodari et al., 2008; Le Danvic et al., 2009).

*In vitro* studies have shown that certain peripheral odorants activate ORs expressed in cultured dopaminergic neurons (Grison et al., 2014). However, these observations do not indicate that the same ligands are used in brain under physiological conditions. Rather, distinct molecules delivered from the blood, cerebrospinal fluid, neighboring local neurons and glial cells, distant cells through the extracellular space and the cells’ own self-regulating internal homeostasis may be postulated as putative ligands in brain.

## Taste Receptors (TASRs)

Taste perception is mediated by taste receptors located in the taste buds located mainly on the tongue. Each taste bud contains between 50 and 100 sensory cells, each with the capacity to react and transduce one of the five categories of taste: bitter, sour, umami, sweet, and salty (Iwatsuki and Uneyama, 2012). Bitter receptors bind to α-gustducin which upon activation opens transient channels producing influx of Na^+^, resulting in cell depolarization (Ishimaru and Matsunami, 2009; Iwatsuki and Uneyama, 2012). α-gustducin is necessary to mediate sweet and bitter taste perception (Wong et al., 1996; Iwatsuki and Uneyama, 2012).

In human, 25 genes encoding bitter taste receptors have been identified (Behrens et al., 2007). As for ORs, ectopic TASRs are localized in many organs and systems (Behrens and Meyerhof, 2010). Recent studies have shown the expression of TASRs and α-gustducin in gastrointestinal tract in both specialized neural cells and selected non-endocrine cells in the mucosa (Höfer et al., 1996; Wu et al., 2002; Dyer et al., 2005; Rozengurt, 2006; Jang et al., 2007; Mace et al., 2007; Margolskee et al., 2007; Egan and Margolskee, 2008; Sternini et al., 2008; Depoortere, 2014; Kokrashvili et al., 2014).

Many TASRs have been related to glucose transport metabolism in which they regulate the expression of specific transporters (Au et al., 2002; Margolskee et al., 2007). Bitter taste receptors are also expressed in the stomach and intestine and their activation produces an increase in the expression of cholecystokinin and peptide YY (Halatchev and Cone, 2005; Chen et al., 2006; Rozengurt et al., 2006). Interestingly, the expression of bitter receptors seems to be partially modulated by diet (Vegezzi et al., 2014).

The role of these receptors in the control of food intake has been elegantly demonstrated by infusion through a nasoduodenal catheter of combinations of tastants; unami alone and the combination of tastants reduced appetite (van Avesaat et al., 2015). Non-caloric sweeteners seem to act on the same sweet receptors and this may explain, at least in part, why diabetes, obesity and metabolic syndrome do not decrease but rather quite the reverse in heavy non-caloric sweetener consumers (Dhingra et al., 2007; Pierce et al., 2007; Lutsey et al., 2008; Davidson et al., 2011; Burke and Small, 2015; Fowler et al., 2015; Pepino, 2015).

Several bitter taste receptors are found in the intestine (Reimann et al., 2012; Kendig et al., 2014; Shirazi-Beechey et al., 2014; Gu et al., 2015). Activation of selected bitter receptors triggers colonic peristaltic movements (Kendig et al., 2014). Also, a complex relationship exists among gut taste receptors, transporters and colonic microbiota (Swartz et al., 2012; Alcock et al., 2014; Kaji et al., 2014).

In contrast to TASRs in the mouth, which transmit information to the brain, TASRs in the digestive system are probably not connected to the brain but rather detect local soluble substances and respond by instructing cells to release hormones and other molecules.

*TASR* genes, their products and down-stream signaling molecules have also been reported in the rat and mouse choroid plexus (Ren et al., 2009; Quintela et al., 2013), where they have been considered as glucose sensors.

Regarding the nervous system, bitter TASRs are expressed in the brain of mice and rats (Singh et al., 2011; Dehkordi et al., 2012; Voigt et al., 2015). *TASR* gene expression has also been identified in the human brain (Ansoleaga et al., 2013; Garcia-Esparcia et al., 2013; Grison et al., 2014). Their function in brain is not known. As for ORs, the term TASRs is misleading when referring to these receptors in the nervous system as they are not related to taste.

## Alterations of Brain ORs (and TASRs) in Parkinson’s Disease (PD)

The concept of Parkinson’s disease has changed with time. First clinically described by J. Parkinson in 1817 in the “Essay of shaking palsy”, it was not until a century later that characteristic neuronal inclusions named Lewy bodies were identified and the substantia nigra was recognized as the main area affected. In the late 60’s of the last century, about 15 years after the discovery of dopamine as the deficient neurotransmitter, the first clinical trials with levodopa (L-DOPA) began to change dramatically the course of the disease. This success, together with the search for pre-clinical symptoms that might help to recognize early stages of the disease and therefore make treatment possible, has had important consequences. Pre-clinical PD should be considered as pre-motor or pre-parkinsonian PD, as several clinical symptoms may precede motor symptoms by many years, including smell disorders, abnormal sleep, variegated neuro-vegetative symptoms, and particular modifications of mood such as apathy and depression. Further, pharmacological and in some cases specific brain stimulation has dramatically increased not only quality of life but also survival. It is not unusual for individuals with PD to live more than 15 years after the clinical diagnosis. Yet this would have other consequences. Since PD is a progressive disease, other brain areas are affected and particular forms of cognitive deterioration, mood disorders, and dementia appear in patients with a long disease course (Ferrer, 2009; Ferrer et al., 2011, 2012; Schneider and Obeso, 2015).

Neuropathological stages of PD have also been established in typical cases. Deposition of aggregated α-synuclein, which is the main component of Lewy bodies and abnormal neurites, first occurs in PD in the olfactory bulb, autonomic nervous system, and selected nuclei of the medulla oblongata at stage 1. Stage 2 involves, in addition, selected nuclei of the pons such as the locus ceruleus. Stage 3 sees spreading to the midbrain and particularly, to the substantia nigra pars compacta, thereby producing dopaminergic denervation in the striatum and other brain regions. Stage 4 involves the limbic system, while stages 5 and 6 progressively compromise the neocortex (Jellinger, 2011; Revesz et al., 2015).

Neuropathological staging apparently supports a structural underpinning to clinical symptoms in PD. Although this is true for pre-parkinsonian and parkinsonian stages of the disease, Lewy bodies that do not properly match with the degree of cognitive impairment and dementia in PD. Alzheimer’s disease (AD) and vascular co-morbidities have been suggested as putative factors (Jellinger, 2011).

About 5–10% of PD cases are familial and inherited with dominant or recessive traits. Autosomal dominant PD has been linked to mutations (but also duplications and triplications) in *SNCA* (the gene encoding α-synuclein), *LRRK2* (encoding leucine-rich repeat kinase 2), and *VPS35* (encoding vacuolar protein sorting 35-homolog). Autosomal recessive PD has been associated with mutations in *PARK2*, encoding parkin, *PINK1* (gene product: PTEN-induced kinase-1), *PARK7* (gene product: DJ1), *ATP13A2* (encoding ATPase type 13A2, *PLAG2G6* (gene product: phospholipase A2 group 6, *FBX07* (gene product F-box protein 7, and *PANK2* (encoding pantothenate kinase 2). Sporadic PD is modulated by several gene variants and undetermined numbers of environmental factors including exogenous pathogens (Houlden and Singleton, 2012; Revesz et al., 2015).

Gene expression analysis followed by qRT-PCR validation in PD has identified a cluster of *OR* genes whose expression is altered in the frontal cortex; these modifications seem to occur at relatively early stages of the disease as they appear at premotor stages and remain in parkinsonian stages of the process (Garcia-Esparcia et al., 2013). *OR2L13*, *OR1E1*, *OR2J3*, *OR52L1* and *OR11H1* are down-regulated. Interestingly, *OR4F4* mRNA shows a significant augmentation only in PD females.

Taste receptors (TASRs) have been analyzed in parallel; *TAS2R5* is down-regulated, and *TAS2R10* and *TAS2R13* up-regulated, at premotor and parkinsonian stages in the same samples. Up-regulation affects mainly males and down-regulation occurs in one gene in females (Garcia-Esparcia et al., 2013). All deregulated TASRs belong to the bitter taste family.

These observations indicate that: (i) cortical ORs and TASRs are vulnerable to PD; (ii) deregulation of selective *OR* and *TASR* genes occurs at relative early stages of the disease; (iii) altered *OR* and *TASR* gene expression is not dependent on treatment because changes are observed in incidental cases discovered on post-mortem neuropathological examination (pre-clinical stages not detected during life) in individuals who did not receive drug therapy; (iv) *OR* gene down-regulation predominates but the expression of some *OR* genes is preserved, and only a few of them are up-regulated; (v) deregulation of *TASR* genes at the same stages of the disease and in the same region is more variable; and (vi) deregulation of certain ORs and TASRs in PD is gender-dependent.

Importantly, functional genomics in the same series has shown deregulation of *UGT2A3*, *UGT2B7* and *UGT2B10* in PD samples, reinforcing the idea of cooperative function with ORs. Interestingly, all these UGTs cluster on chromosome 4q13.

Regarding the substantia nigra, *OR2L13*, *OR2T33*, *OR2J3*, *OR52L1*, *OR10G8*, *OR11H1* and *OR4F4* are down-regulated in PD (Grison et al., 2014). Decreased expression of all these receptors might be interpreted as the consequence of neuron loss in this region. However, *TAS2R4*, *TAS2R5* and *TAS2R50* mRNA expression is increased in the same samples (Grison et al., 2014). Therefore, altered expression of ORs and TASRs in the substantia nigra appears to be highly regulated in PD.

## Alterations of Brain ORs (and TASRs) in Alzheimer’S Disease (AD) and Related Mouse Model

The concept of AD has been modified over the years. The seminal definition first advanced by A. Alzheimer was of a form of pre-senile dementia (appearance of the clinical symptoms before the age of 65 years) characterized by the accumulation of extracellular structures named senile plaques and intra-neuronal tangles of fibrils visualized with specific silver stains. These changes were similar to those seen in a number of cases with dementia appearing before the age of 65 years and identified as senile dementia (of Alzheimer’s type). Senile plaques composed of a core of amyloid together with neurofibrillary tangles composed of hyper-phosphorylated and aggregated tau have been the hallmarks of AD regardless of the age at onset of clinical symptoms. We now know that early onset AD (EOAD) is often inherited and linked to mutations in the three genes *APP*, *PSEN1*, and *PSEN2*, encoding β-amyloid precursor protein, presenilin 1, and presenilin 2, respectively (familial EOAD: EOFAD), whereas late onset AD (LOAD) is currently sporadic and modulated by increased numbers of genetic factors with low penetrance (genetic modulators or variants) particularly APOEε4, and environmental factors (Bertram and Tanzi, 2011; Duyckaerts and Dickson, 2011; Serrano-Pozo et al., 2011; Lowe and Kalaria, 2015).

Like other neurodegenerative diseases with abnormal protein aggregates, AD is a long-lasting biological process in which only the advanced stages of the disease manifest with cognitive impairment and dementia. Therefore, AD is now classified clinically as prodromic or preclinical (without symptoms), mild cognitive impairment of AD origin, and AD (dementia of AD). Tremendous efforts are now underway to identify early biomarkers of AD in living individuals (Knopman, 2011).

Additionally, neuropathological studies of consecutive autopsy cases in young, middle-aged, and elderly individuals with and without cognitive impairment have shown a progression in the localization, distribution, and extent of β-amyloid plaques and neurofibrillary tangles in the non-selected population. Based on these neuropathological findings, the most widely accepted neuropathological classifications divide AD into stages I, II, III, IV, V, and VI depending on the neurofibrillary tangle pathology (Braak and Braak, 1991, 1999), and into stages 0, A, B, and C (Braak and Braak, 1999), or 0, 1, 2, 3, and 4 (Thal et al., 2002) depending on the distribution of senile plaques or β-amyloid deposits.

Following the Braak and Braak nomenclature, stages I–II refer to neurofibrillary tangle involvement of the trans-entorhinal and entorhinal cortices; stages III–IV include additional entanglement of the hippocampus, deep temporal cortex, and limbic regions; and stages V–VI manifest with large numbers of neurofibrillary tangles in the neocortex eventually including primary sensory areas. First stages are subclinical and they can last for decades whereas mild cognitive impairment and dementia occur at stages IV and VI (for review see Ferrer, 2012).

To be more precise, certain individuals in their thirties already show the early changes of AD. About 85% of individuals aged 65 years have AD at stages I–II at least, but only 5% have extended lesions clinically manifested as dementia. Cortical involvement increases with age and about 25% of the population has Alzheimer’s dementia at the age of 85 years (Braak et al., 2011; Ferrer, 2012).

A region on 14q11.2, between 19.2 and 19.49 MB, is linked to earlier age of the appearance of clinical symptoms, as revealed using array comparative genome hybridization and replicated evaluation with Affymetrix array and multiplex ligation-dependent probe amplification (Shaw et al., 2011). This association is independent of and in addition to the association observed for APOE4. The association signal comes from olfactory receptor gene cluster in the region where *OR4M1*, *OR4N2*, *OR4K2*, *OR4K5* and *OR4K1* are located. The 2nd copy and 3rd copy states have similar age at onset and the 4th copy state has a trend toward earlier age at onset on all APOE4 backgrounds. The 5 + copy subjects were 2 times more likely to be diagnosed with AD by any given age compared to the 2, 3, and 4 copy carriers. The risk of onset before age 72 years for persons who had 5 copies or more was 6-fold higher compared to persons having lower copy numbers (Shaw et al., 2011).

*OR* gene expression in AD has been studied in the entorhinal cortex and frontal cortex area 8 covering all the stages of disease progression. *OR11H1* mRNA is increased and *OR4F4* mRNA decreased in the AD entorhinal cortex at stages III–IV and V–VI compared with controls. *OR10G8* mRNA expression levels decrease in the entorhinal cortex at late stages of AD (Ansoleaga et al., 2013). *OR4F4* mRNA expression levels are increased in the frontal cortex area 8 at stages III–IV, and *OR52L1* mRNA at stages III–IV and V–VI (Ansoleaga et al., 2013).

Whether changes in AD can be replicated in transgenic models of AD has been tested in APP/PS1 transgenic mice which bear the Swedish *APP* (K594M/N595L) mutation and the dE9 deletion in *PSEN1* (Borchelt et al., 1996; Aso et al., 2012). APP/PS1 mice were developed by co-injection of the two transgene constructs [mouse/human (Mo/Hu) chimeric APP695 harboring the Swedish (mutation and exon-9-deleted PS1] into pronuclei with a single genomic insertion site resulting in the two transgenes being transmitted as a single mendelian locus (Borchelt et al., 1996).

APP/PS1 transgenic mice exhibit alterations in the central OR signaling pathways. *Olfr110* mRNA, the only OR assessed, significantly increases from the age of 3 months onwards, whereas *Adcy3* mRNA expression levels are reduced in transgenic mice aged 12 months (Ansoleaga et al., 2013).

Regarding TASRs, increased *TAS2R13* mRNA expression is observed at stages III–IV, and *TAS2R5* and *TAS2R10* mRNA expression at stages V–VI in the entorhinal cortex. Yet no modification in the mRNA expression levels of these TASRs is observed in frontal cortex area 8 at any stage of AD (Ansoleaga et al., 2013).

## Alterations of Brain ORs (and TASRs) in Progressive Supranuclear Palsy (PSP)

PSP is a neurodegenerative disease characterized clinically by pseudobulbar palsy, vertical ocular palsy, nuchal dystonia, and dementia. The term was first coined as PSP following description by J.C. Steele, J.C. Richardson, and J. Olszewski in the mid-60’s of the last century. The main pathological finding is the accumulation of abnormally hyper-phosphorylated 4 R tau in neurons in the form of pre-tangles and tangles, astrocytes forming characteristic tufts, and oligodendrocytes in the form of coiled bodies in several brain regions including selected nuclei of the brain stem, diencephalic nuclei, cerebellum, and several areas of the telencephalon. PSP has rarely been reported as a familial disease, presenting most commonly as a sporadic form (Dickson et al., 2011).

The same *OR* genes assessed in PD and AD have been assessed in the frontal cortex (area 8) of PSP at the terminal stage of the disease. *OR11H1*, *OR2D2*, *OR2L13*, *OR2D2*, *OR2T33*, *OR4F4*, *OR10G8* and *OR52L1* mRNAs are up-regulated whereas *OR51E1* mRNA levels are similar to those of controls (Ansoleaga et al., 2013). Interestingly, all mRNAs of TASRs analyzed, *TAS2R4*, *TAS2R5*, *TAS2R10*, *TAS2R13*, *TAS2R14* and *TAS2R50*, are up-regulated in the same cortical region in PSP (Ansoleaga et al., 2013).

## Alterations of Brain ORs (and TASRs) in Sporadic Creutzfeldt-Jakob Disease (sCJD)

CJD was first described at the beginning of the second decade of the last century by H. G. Creutzfeldt and A.M. Jakob but only two seminal cases are now considered real CJD. The disease is manifested clinically as rapid dementia plus ataxia, myoclonus, and other neurological symptoms, and neuropathologically as spongiform encephalopathy with neuron loss, spongiform degeneration (small and often confluent brain vacuoles), and microglia and astroglia activation. CJD is the paradigm of human prion diseases rarely caused by mutations in *PRNP*, the gene encoding the prion protein (fCJF; about 10% of cases), transmission of abnormal prion from one individual to another (iCJD; very rare but most commonly represented by the variant Crreutzfeldt-Jakob disease (vCJD) resulting from the transmission of bovine spongiform encephalopathy-contaminated food), or apparently occurring de novo as sporadic CJD (sCJD, representing the most common form albeit having an incidence of about 1–2 cases per 1,000,000 inhabitants per year). In all these circumstances, the normal prion protein is transformed into an abnormal, predominantly but not exclusively proteinase-resistant form called a prion, which is pathogenic. PrP presents in sporadic cases as type I and type II depending on the molecular weight of the non-glycosylated isoform and the amount of the mono- and di-glycosylated isoforms. Interestingly, CJD is also conditioned by the characteristics of codon 129 in *PRNP* (methionine/methionine, valine/valine, and methionine/valine) which marks the phenotypes of fCJD, iCJD, and sCJD. Focusing on sCJD, several types may be categorized according to the codon 129 in *PRNP* and prion type; the most frequent are MM1 and VV2 (Head and Ironside, 2009; Parchi et al., 2009, 2011; Zerr et al., 2009; Budka et al., 2011).

Frontal cortex area 8 has been assessed in sCJD subtypes MM1 and VV2 to investigate regional commonalities and differences between the two main subtypes of sCJD. Involvement of the cerebral cortex predominates in subtype MM1 whereas the cerebellum is severely affected in subtype VV2. PrP^Res^ deposition differs in the two subtypes: a synaptic pattern occurs in sCJD MM1 and perineuronal deposits characterize sCJD VV2 in the cerebral cortex, while PrP^Res^-plaques are common in the cerebellum in sCJD VV2 (Budka et al., 2011).

*OR2L13, OR4F4*, *OR51E1* and *OR52L1* mRNA expression is reduced in the frontal cortex in sCJD MM1; *OR11H1* mRNA is down-regulated in sCJD MM1 and VV2; and *OR10G8* mRNA expression levels are significantly increased in sCJD VV2 (Ansoleaga et al., 2013). Regarding the cerebellum, *OR2D2* is up-regulated in sCJD MM1, while *OR4F4* and *OR51E1* mRNA decrease in sCJD MM1 and VV2, respectively (Ansoleaga et al., 2013).

*TAS2R4*, *TAS2R10* and T*AS2R14* mRNA expression is increased in the frontal cortex area 8 in sCJD MM1 and VV2, but increased *TAS2R5* mRNA is only in MM1, and *TASR13* mRNA only in VV2 (Ansoleaga et al., 2013).

## Comparative Aspects of OR and TASR mRNA Expression in Frontal Cortex Area 8 in PD, AD, PSP and sCJD

The study of gene expression performed in the same cortical region (frontal cortex area 8) using the same probes, the same method and the same hands in the same laboratory permits a comparative evaluation of the results obtained in PD, AD, PSP and sCJD subtypes MM1 and VV2. Curiously, AD shows little modification when compared with the other diseases. In contrast, *OR* gene up-regulation occurs in 8 of 11 analyzed *OR* genes. Predominant OR mRNA down-regulation is found in sCJD mainly in subtype MM1. Finally, reduced mRNA expression of several ORs occurs in the frontal cortex area 8 in PD in males but up-regulation of one OR occurs in females (Table 1).

**Table 1:** **Comparative aspects of olfactory (odorant) receptors (*ORs*) and taste receptors (*TASR*) gene expression in frontal cortex area 8 in Parkinson’s disease (PD), Alzheimer’s disease (AD) II–IV and V–VI corresponding stages of Braak and Braak, Progressive Supranuclear Palsy (PSP) and sporadic Creutzfeldt-Jakob disease (sCJD) subtypes MM1 and VV2, from observations described in reference Ansoleaga et al. (2013) and Garcia-Esparcia et al. (2013)**.

	PD	AD		PSP	sCJD	
		III/IV	V/VI		MM1	VV2
*OR2D2*	=	=	=	↑	=	=
*OR2J3*	↓ only males	ND	ND	ND	ND	ND
*OR2L13*	T↓	=	=	↑	↓	=
*OR2T33*	=	=	=	↑	=	=
*OR4F4*	↑ only females	↑	=	↑	↓	=
*OR10G8*	ND	=	=	↑	=	↑
*OR11H1*	T↓; males ↓	=	=	↑	↓	↓
*OR51E1*	T↓; males ↓	=	=	=	↓	=
*OR52L1*	T↓; males↓	↑	↑	↑	↓	=
*TAS2R4*	=	=	=	↑	↑	↑
*TAS2R5*	↓; females	=	=	↑	↑	=
*TAS2R10*	↑; males ↑	=	=	↑	↑	↑
*TAS2R13*	↑; males ↑	=	=	↑	=	↑
*TAS2R14*	=	=	=	↑	↑	↑
*TAS2R50*	=	=	=	↑	=	=

T, total; ND, not done; =, no significant difference compared with controls.

Parallel studies of TASR mRNA expression reveal no significant differences in the expression of analyzed genes in AD, up-regulation of six of the six analyzed genes in PSP, predominant up-regulation in sCJD, and increased or depressed mRNA expression of *TASR* genes depending on gender in PD (Table 1).

These observations show differential regulation of *OR* and *TASR* genes in frontal cortex area 8 in PD, AD, PSP and sCJD. These changes cannot be attributed merely to cell death, as some genes are up-regulated, others are down-regulated and still others show no modifications when compared with gene expression in control individuals.

Olfactory signaling in olfactory organs and smell perception are subject to gender differences (Stowers and Logan, 2010). This may be related to distinct and complementary factors such as defined gender responses to sex-specific initial signals (Holy et al., 2000; Luo et al., 2003; Ben-Shaul et al., 2010; Haga et al., 2010) probably secondary to modulation by steroid hormones (Leypold et al., 2002; Stowers et al., 2002; Kimchi et al., 2007; Raskin et al., 2009; Wu et al., 2009; Juntti et al., 2010). The observations in PD indicate that at least a sub-population of cortical cerebral ORs and TASRs in PD is modulated by sex-specific initial signals, sexually-dimorphic specific ligands or steroid hormones and their derivatives (Garcia-Esparcia et al., 2013). No similar data are available for AD, PSP or CJD.

## ORs (and TASRs) in the Cerebral Cortex in Schizophrenia

mRNA levels of *OR2T1, OR2T33*, *OR52H1, OR2D2, OR10G8, OR51E1* and *OR52L1* are significantly reduced in the prefrontal cortex of patients with schizophrenia. The main changes cluster in 11p15.4, 1q44 and 11q24.2 (Ansoleaga et al., 2015). *TAS2R4*, *TAS2R13, TAS2R5* and* TAS2R50* mRNAs are decreased in the prefrontal cortex in the same group of patients (Ansoleaga et al., 2015).

Gene deregulation does not correlate with negative symptoms or with the duration of the disease, but mRNA expression levels of olfactory receptors *OR2T1, OR252L1*, *OR2D2, OR10G8*, and taste receptors *TAS2R5, TAS2R13*, and *TAS2R50* show an association with treatment with antipsychotics, particularly chlorpromazine (Ansoleaga et al., 2015).

These findings show that brain deregulation of ORs and TASRs is not restricted to neurodegenerative diseases with abnormal protein aggregates but rather deregulation can also occur in neuropsychiatric disease. Moreover, modulation of certain ORs and TASRs largely depends on antipsychotics in schizophrenia, thus widening the scenario of OR and TASR regulation to variegated pharmacological agents (Ansoleaga et al., 2015).

## Final Comments

ORs in regions other than those located in specific olfactory sensory organs have been categorized as “ectopic” ORs. These receptors are widely distributed in many organs in mammals where they play distinct functions not directed to the perception of odors as they are not connected to olfactory centers in brain. In most regions, ectopic ORs await identification of putative ligands and physiological functions. However, the impressively large number of genes and the wide distribution in their expression support a cardinal role for ORs (and TASRs) in homeostasis, possibly with the complementary action of different receptors.

Most work on Gαolf in human brain has been related to its expression in the striatum, its relation with dopamine D1 and adenosine A2A receptors (Corvol et al., 2001; Hervé, 2011) and its possible implications in movement disorders and mental disorders and in animal models in which dopamine is implicated. Gαolf is regulated by dopamine in the striatum, which may have implications in PD (Ruiz-DeDiego et al., 2015). In this line, certain polymorphisms in *GNAL* have been linked to bipolar and attention deficit/hyperactivity disorders (Berrettini et al., 1998; Laurin et al., 2008). Gαolf protein levels in rat striatum are increased by chronic antidepressant treatment (Taoka et al., 2006). Mutations in *GNAL*, which encodes Gαolf, are causative of craniocervical dystonia (Fuchs et al., 2013; Vemula et al., 2013; Kumar et al., 2014).

Gαolf and AC3 are not specific to the OR down-stream signaling pathway as they are used in multiple intracellular signaling processes.

Even considering that almost half of the human OR repertoire is probably non-functional (Rouquier and Giorgi, 2007; Go and Niimura, 2008), alterations in gene expression which are differentially regulated in a number of neurodegenerative and mental human diseases make the scenario a subject of attention. This is further driven by the fact that observations in other organs and systems have shown that ORs located in non-olfactory organs are chemoreceptors with unexpected functions.

The presence of a variable number of ORs in a single neuron, the variety of putative odorant ligands and the different feasible responses dependent on the region where the neuron is located and operates make elucidating the function of ORs in the nervous system an apparently tremendous task. The study of single receptors expressed in neural cell lines and combined receptors in particular primary neuron cultures in combination with screening of putative agonists of ORs and suspected OR ligands, most of them probable intrinsic metabolites, will facilitate the task. Design and isolation of small molecules to act on selected ORs functioning as cell-type specific agonists or antagonists seems a promising tool. Reconstruction of the tri-dimensional structure of receptors will help in the analysis of mechanisms of activation and in the recognition and design of functional ligands, and specific agonists and antagonists (Persuy et al., 2015; Wolf and Grünewald, 2015).

The activation of ORs and immune cellular responses by common molecules in several systems (Jang et al., 2013; Li et al., 2013b; Perricone et al., 2013; Stopková et al., 2014; Ward et al., 2015), and the cooperation of certain MHC genes in the expression of ORs in the vomeronasal organ of mice (Leinders-Zufall et al., 2014), lend support to the working hypothesis that the same scenario may occur in brain, where the central olfactory system expressed in neurons and the brain immune system involving microglia, astrocytes and neurons work in cooperation. The fact that the CNS is not an isolated immune organ but rather interacts with the systemic immune system, together with the expression of ORs and related OR factors in brain and choroid plexus directly interacting with blood, reinforces the notion that ORs in the CNS play novel functions unrelated to olfaction.

## Author Contributions

IF: designed and wrote the article, PG-E and MC: carried out immunohistochemistry; EC: studied the choroid plexus; EA and GGK: studied the developing brain; AG and SG: participate in the discussion and criticism of the manuscript.

## Conflict of Interest Statement

The authors declare that the research was conducted in the absence of any commercial or financial relationships that could be construed as a potential conflict of interest.

## Abbreviations

*AC3*/AC3: adenylyl cyclase 3 gene and protein, human
AD: Alzheimer’s disease
*Adcy3*: adenylate cyclase 3 gene, mouse
APOE4: apolipoprotein 4
APP: amyloid precursor protein
cAMP: cyclic adenosine monophosphate
Golf: olfactory G protein composed of three subunits: α, β and γ
Gαolf: olfactory G protein α subunit
*GNAL/gnal*: guanine nucleotide binding protein (G protein), alpha activating activity polypeptide olfactory type gene, human and mouse respectively, which encodes Gαolf
GPCRs: G-protein-coupled receptors
OBP: olfactory-binding protein
*Olfr13* (MOR23), *Olfr110*, *Olfr287*: names of olfactory receptor genes, mouse
OMP: olfactory marker protein
ORs: olfactory (odorant) receptors
*OR2D2*, *OR2H2*, *OR2J3*, *OR2L13*, *OR2T33*, *OR4F4*, *OR10G8*, *OR11H1*, *OR51E1*, *OR52L1*: names of different human olfactory receptor genes
OR2H2, OR2A4, OR6K3: names of olfactory receptor proteins
PD: Parkinson’s disease
PrP^Res^: Prion protein resistant to proteinase K, Prion
PSEN1: Presenilin 1
PSP: Progressive supranuclear palsy
RTP1 and RTP2: Receptor Transporter Proteins 1 and 2
REEP1: Receptor Expression Enhancing Protein 1
sCJD: sporadic Creutzfeldt-Jakob disease
subtypes MM1: Methionine/methionine at codon 129 of *PRNP*
VV2: valine/valine at codon 129 of *PRNP*
*PRNP*: prion protein gene
TASR: taste receptors
*TAS2R4*, *TAS2R5*, *TAS2R10*, *TAS2R13*, *TAS2R14*, *TAS2R50*: names of different human taste receptor genes
UGTs: UDP-glucuronosyltransferases
*UGT1A6*, *UGT2A3*, *UGT2B10*: names of different human UGT genes.
